# Cincinnati Dental Association

**Published:** 1859-07

**Authors:** J. Richardson

**Affiliations:** Cincinnati


### Proceedings of Societies.

### CINCINNATI DENTAL ASSOCIATION.

CINCINNATI, May 10, 1859.

Dr. BONSAL related case of boy, æt. twelve years, who had been struck with a bat, fracturing the processes and partially dislodging the right superior center and lateral incisor teeth. These teeth were forced from their sockets by the blow, and found hanging loosely with their cutting edges over half an inch below the circle of the adjoining teeth. Softened gutta percha, moulded into proper form, was pressed against the displaced teeth, carrying them back to their proper place, and were retained *in situ* by a bandage passing underneath the chin and over the head. This was removed, from time to time, to permit the patient to take food and drink, and was worn for two weeks and removed. At the end of about one month the parts had healed without any unpleasant sequellæ, and the teeth acquired their usual firmness. Dr. B. could not state, not having seen the patient very recently, what amount of discoloration, if any, had taken place in the injured teeth.

Dr. IRWIN also reported case of man about twenty years old, with anterior displacement of the inferior incisor teeth with fracture of corresponding portion of the anterior walls of the processes. The patient, in a personal encounter, had seized hold of his adversary's finger with his teeth, and the latter in attempting to relieve himself, wrenched the lower incisors from their sockets, carrying with them the anterior processes. The teeth and fractured portions of the jaw were pressed back into the circle and retained in place by ligatures attached to the molar teeth. The parts united by first intention and healed kindly within a few weeks.

Dr. Taft expressed continued confidence in the use of nitric acid as an application to exposed nerves, preparatory to filling. Had treated some fifteen or more cases with invariable success.

Dr. Richardson had also experimented with same remedy, with satisfactory results.

A case of exalted sensibility of dentine, after filling, was related by Dr. Taft, the details of which will be found in present number, page 10.

J. Richardson, Sec'y.

